# Chloroquine inhibits human CD4^+^ T-cell activation by AP-1 signaling modulation

**DOI:** 10.1038/srep42191

**Published:** 2017-02-07

**Authors:** Ralf L. J. Schmidt, Sabrina Jutz, Katrin Goldhahn, Nadine Witzeneder, Marlene C. Gerner, Doris Trapin, Georg Greiner, Gregor Hoermann, Guenter Steiner, Winfried F. Pickl, Heinz Burgmann, Peter Steinberger, Franz Ratzinger, Klaus G. Schmetterer

**Affiliations:** 1 Department of Laboratory Medicine, Medical University of Vienna, Austria; 2 Institute of Immunology, Center for Pathophysiology, Infectiology and Immunology, Medical University of Vienna, Austria; 3 Division of Rheumatology, Department of Internal Medicine III, Medical University of Vienna, Austria; 4 Cluster Arthritis and Rehabilitation, Department of Degenerative Joint Diseases, Ludwig Boltzmann Society, Vienna, Austria; 5 Department of Medicine I, Division of Infectious Diseases and Tropical Medicine, Medical University of Vienna, Vienna, Austria

## Abstract

Chloroquine (CQ) is widely used as an anti-inflammatory therapeutic for rheumatic diseases. Although its modes of action on the innate immune system are well described, there is still insufficient knowledge about its direct effects on the adaptive immune system. Thus, we evaluated the influence of CQ on activation parameters of human CD4^+^ T-cells. CQ directly suppressed proliferation, metabolic activity and cytokine secretion of T-cells following anti-CD3/anti-CD28 activation. In contrast, CQ showed no effect on up-regulation of T-cell activation markers. CQ inhibited activation of all T helper cell subsets, although IL-4 and IL-13 secretion by Th2 cells were less influenced compared to other Th-specific cytokines. Up to 10 μM, CQ did not reduce cell viability, suggesting specific suppressive effects on T-cells. These properties of CQ were fully reversible in re-stimulation experiments. Analyses of intracellular signaling showed that CQ specifically inhibited autophagic flux and additionally activation of AP-1 by reducing phosphorylation of c-JUN. This effect was mediated by inhibition of JNK catalytic activity. In summary, we characterized selective and reversible immunomodulatory effects of CQ on human CD4^+^ T-cells. These findings provide new insights into the biological actions of JNK/AP-1 signaling in T-cells and may help to expand the therapeutic spectrum of CQ.

The antimalarial drugs chloroquine (CQ) and hydroxy-chloroquine (HCQ) are disease-modifying antirheumatic drugs (DMARD)^1^,^2^, which are used in the therapy of rheumatic and connective tissue diseases, including systemic lupus erythematosus and rheumatoid arthritis^3^,^4^,^5^. Especially in patients with methotrexate (MTX) failure, the combination of CQ or HCQ with MTX has an efficacy similar to that of the combination of MTX with biologicals^6^,^7^. Furthermore, these 4-aminoquinoline derivatives have a favorable drug safety profile, with retinal toxicity as their main adverse event.

The immunosuppressive potency of CQ has been mainly attributed to its properties as a weak lipophilic base, which highly enriches in acidic intracellular vesicles such as lysosomes. These lysosomotropic kinetics result in the modulation of multiple processes which affect immune cell functions. First, the de-acidification of endolysosomes strongly impairs the antigen processing capacity of monocytes and dendritic cells, thereby suppressing antigen presentation to CD4^+^ T-cells^8^,^9^,^10^. By similar mechanisms, CQ also decreases the signaling of intracellular toll-like receptors^11^,^12^. Furthermore, lysosomal accumulation of CQ inhibits autophagy processes, which may also contribute to the immunomodulatory properties of CQ^13^,^14^. The adaptive immune system and especially T-cells are critically involved in the pathogenesis of rheumatic and connective tissue diseases^15^. Thus, beneficial effects of CQ might also be attributed to direct modulation of T-cells. Notably, there is little evidence available regarding the direct effects of CQ on T-cell function^16^. Decreased lymphocyte proliferation and IL-2 mRNA production in CQ-exposed T-cells were first described by seminal studies^17^,^18^. On the molecular level, inhibition of calcium signaling by chloroquine has been reported in murine thymocytes and the human Jurkat T-cell line^19^,^20^. However, methodological differences, including the types of cells evaluated, parameters measured and especially CQ concentrations used, do not allow a definite conclusion to be drawn, and a comprehensive overview of the direct effects of CQ on CD4^+^ T-cells is still lacking.

Consequently, we assessed the effects of CQ on parameters of T-cell function, including proliferation, cytokine secretion, autophagy and viability. Further, major pathways of T-cell activation were studied by use of Jurkat reporter cell lines, intracellular flow cytometry, immunoblotting and phospho-protein-specific ELISA and *in vitro* kinase assays.

## Results

### Effects of CQ on the activation of CD4^+^ T-cells

**Figure 1:**
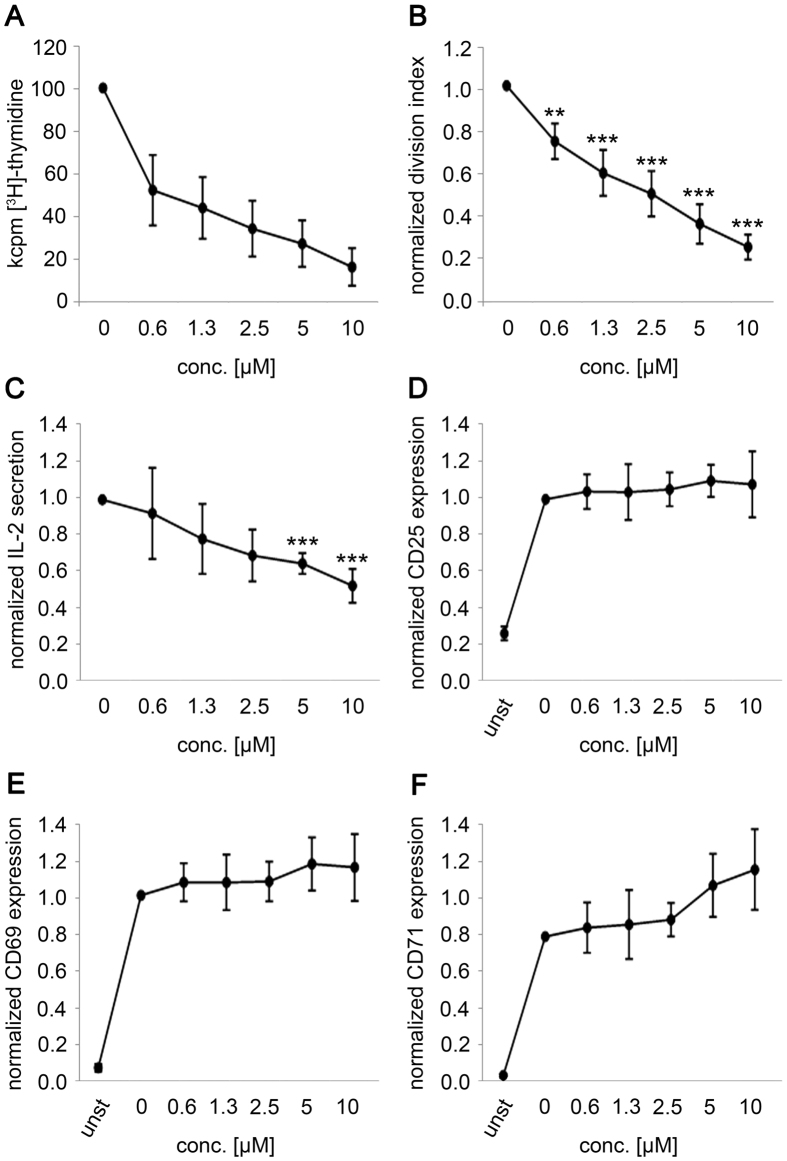
Modulation of T-cell activation parameters by CQ. Human CD4^+^ T-cells were pre-incubated with the indicated concentrations of CQ and activated with anti-CD3/anti-CD28 antibodies. (**A**) Results from thymidine incorporation assays; data depict mean ± SD from triplicate cultures from one representative donor (n = 6) (**B**) Normalized division indices 96 hours after activation from CPD-dilution experiments (n = 6) (**C**) Normalized IL-2 secretion values from supernatants 24 hours after activation (n = 6) (**D**–**F**) Normalized expression of CD25 (**D**), CD69 (**E**) and CD71 (**F**) 24 hours after activation (n = 4). (**B**–**F**) Data show mean ± SD normalized to the value in drug-free medium (0 μM) of the respective donor. *p < 0.05; **p < 0.01; ***p < 0.001.

In thymidine incorporation assays, CQ suppressed T-cell proliferation in a dose-dependent manner. A significant reduction of proliferation was already found at 0.6 μM CQ (0.52 ± 0.17 normalized proliferation rate for CQ, p < 0.001; Fig. 1A) and reached 0.15 ± 0.09 at 10 μM CQ. This finding was confirmed in a second proliferation assay using dilution of a fluorescent cell proliferation tracker (Fig. 1B). IL-2 secretion, representing an early activation read-out, was also reduced beginning with 2.5 μM CQ (p = 0.041, Fig. 1C). In contrast to the parameters described above, the up-regulation of the T-cell activation markers CD25, CD69 and CD71 was not suppressed by CQ (Fig. 1D–F and Supplementary Figure I). For CD71, a trend towards slight up-regulation could be observed, which was more pronounced at high concentrations, but did not reach statistical significance (10 μM CQ: 1.48 ± 0.2; p = 0.173).

### Effects of CQ on the viability and re-stimulation capability of activated CD4^+^ T-cells

**Figure 2:**
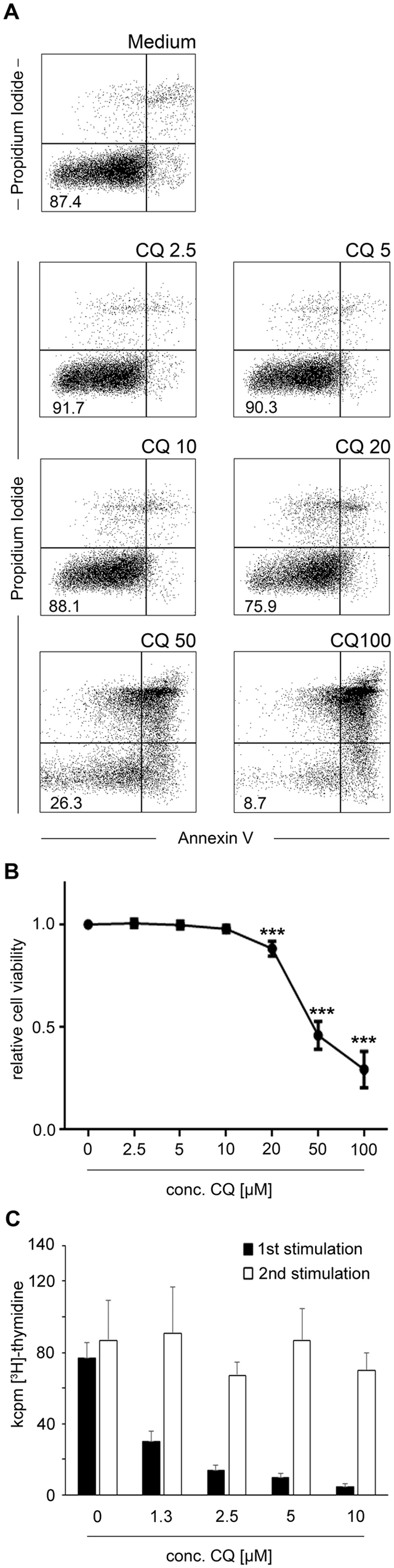
Cell viability and restimulatory capacity of CQ-exposed T-cells. CD4^+^ T-cells were activated in the presence or absence (Medium) of the indicated concentrations of CQ and activated with anti-CD3/anti-CD28 antibodies. After 72 hours, cell viability was measured by Annexin V (x-axis) and Propidium Iodide (y-axis) staining. (**A**) Dot-plots from one representative donor. Numbers indicated percentage of viable (Annexin V^−^/PI^−^) cells. (**B**) Cumulative data from eight healthy donors. (**C**) CD4^+^ T-cells were activated in the presence or absence (0 μM) of the indicated concentrations of CQ and thymidine incorporation was measured after 96 hours (black bars). After seven days, CQ was removed and cells were restimulated for another 96 hours in CQ-free medium (white bars). Data depict mean + SD from triplicate cultures from one representative donor (n = 5). ***p < 0.001.

Up to a concentration of 10 μM, CQ did not affect viability of activated T-cells (p = 0.127). Starting with 20 μM CQ, a significant decrease in T-cell viability was observed (normalized mean: 0.88; p = 0.003), which further decreased to 0.29 ± 0.10 at 100 μM CQ (p < 0.001; Fig. 2A,B). The long-term influence of CQ on T-cell function was simulated by re-stimulation experiments with T-cells pre-cultured in CQ for seven days. As described above (Fig. 1A,B), a CQ-mediated dose-dependent inhibition of T-cell proliferation was found during the primary stimulation. In contrast, T-cells were fully responsive following secondary stimulation, irrespective of the CQ concentration used during first stimulation (Fig. 2C).

### Effects of CQ on metabolic activity

**Figure 3:**
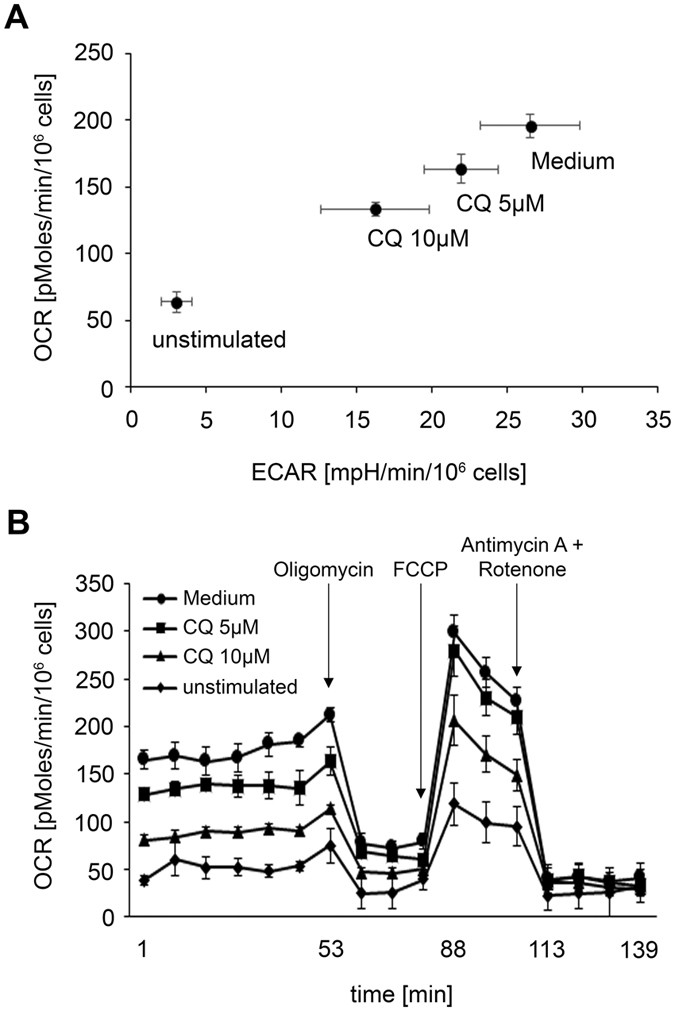
Metabolism in CQ-exposed T-cells. CD4^+^ T-cells were activated in the presence or absence (Medium) of the indicated concentrations of CQ and activated with anti-CD3/anti-CD28 antibodies or left unstimulated. After 48 hours, the extracellular acidification rate (ECAR; representing anaerobic glycolysis) and the oxygen consumption rate (OCR; representing mitochondrial respiration) were measured. (**A**) Mean ± SD from one representative donor (n = 5) (**B**) Mitochondrial stress test; At the indicated time points (arrows), the ATP coupling reagent oligomycin, the electron transfer chain uncoupling reagent FCCP, and the mitochondrial inhibitors rotenone/antimycin A were added. Data depict mean ± SD from quintuplet cultures from one representative donor (n = 5).

T-cell activation is accompanied by profound changes in metabolism, including increased mitochondrial respiration and anaerobic glycolysis, which can be measured by the oxygen consumption rate (OCR) and the extracellular acidification rate (ECAR) respectively^21^. In accordance with the activation parameters described above, exposure to 5 μM or 10 μM CQ also significantly suppressed up-regulation of the basal OCR (p = 0.034 and p = 0.002 respectively) and the ECAR (p = 0.025, p = 0.011, Fig. 3A) following activation. A linear reduction of the OCR/ECAR ratio was found, indicating that CQ affected mitochondrial respiration and anaerobic glycolysis to a similar extent. To further define the influence of CQ on mitochondrial respiration, we performed stress test analyses, where sequential addition of the ATP coupling reagent oligomycin, the electron transfer chain uncoupling reagent FCCP and the mitochondrial inhibitors rotenone/antimycin A allows to dissect parameters of mitochondrial respiration such as the ATP turnover rate, the spare and maximal respiratory rate and the non-mitochondrial respiration. These experiments revealed that ATP turnover rate and maximal respiratory capacity were reduced in CQ-exposed T-cells (p < 0.05 each; Fig. 3B).

### Effects of CQ on cytokine secretion on total T-cells and Th-subsets

**Figure 4:**
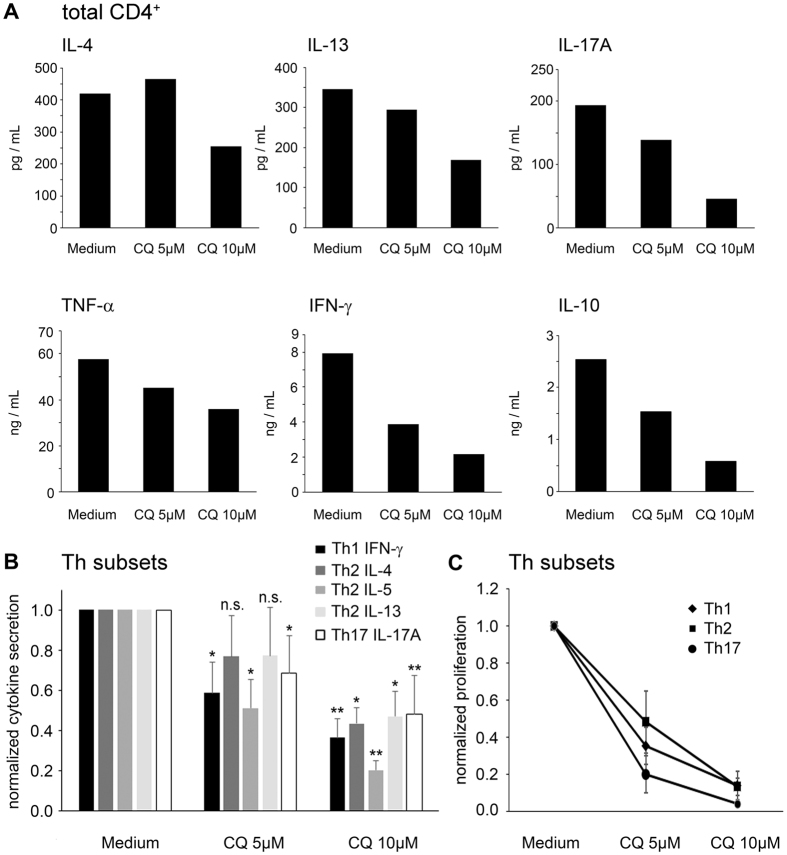
Cytokine production in CQ-exposed T-cells. Total CD4^+^ T-cells or FACS-sorted T helper cells were exposed to the indicated concentrations of CQ or CQ-free medium and activated with anti-CD3/anti-CD28 antibodies. After 72 hours, supernatant levels of the indicated cytokines were measured. (**A**) Supernatant concentrations from total CD4^+^ T-cells. Data show mean values from duplicate measurements from one representative donor (n = 8). (**B**) Normalized cytokine secretion of FACS-sorted Th1 (CD4^+^CD45RO^+^CXCR3^+^), Th2 (CD4^+^CD45RO^+^CCR6^-^CCR4^+^) and Th17 (CD4^+^CD45RO^+^CCR6^+^CCR4^+^) cells. Data show mean + SD from four healthy donors. (**C**) Normalized proliferation of FACS-sorted Th1, Th2 and Th17 cells measured by [^3^H]-thymidine incorporation. Data show mean ± SD from four healthy donors. n.s. not significant; **p < 0.05; **p < 0.01.

Potential Th-specific effects of CQ were evaluated by analysis of the cytokine secretion profile. Effector cytokine levels without subset specificity (TNF-α: 0.62 ± 0.18, IL-10: 0.30 ± 0.15, normalized mean ± SD), as well as Th1-derived (IFN-γ: 0.38 ± 0.15, normalized mean) and Th17-derived (IL-17: 0.32 ± 0.06) cytokines, were strongly decreased by 10 μM CQ. In contrast, the inhibition of Th2 cytokines was less pronounced (IL-4: 0.59 ± 0.20, IL-13: 0.63 ± 0.13, Fig. 4A). Accordingly, at 5 μM CQ, no inhibition of IL-4 and IL-13 secretion was found, while all other effector cytokines measured were significantly suppressed (Fig. 4A and Table 1).

To define these effects in more detail, we FACS-sorted Th1, Th2 and Th17 cells (purity: >95%) according to their highly specific chemokine receptor expression profiles (Supplementary Figure II). As above, secretion of IFN-γ by Th1 cells and of IL-17 by Th17 cells was strongly decreased by CQ (Fig. 4B). IL-4 and IL-13 secretion by Th2 cells was markedly decreased at 10 μM CQ, while exposure to 5 μM CQ affected these cytokines to a lesser degree. In contrast, IL-5 levels from Th2 cells were already significantly reduced at 5 μM CQ (0.51 ± 0.14; p = 0.041), an effect which was even more pronounced at 10 μM CQ (0.20 ± 0.05; p = 0.002, Fig. 4B). However, proliferation of all Th subsets was equally inhibited by CQ (Fig. 4C).

### Effects of CQ on autophagy

**Figure 5:**
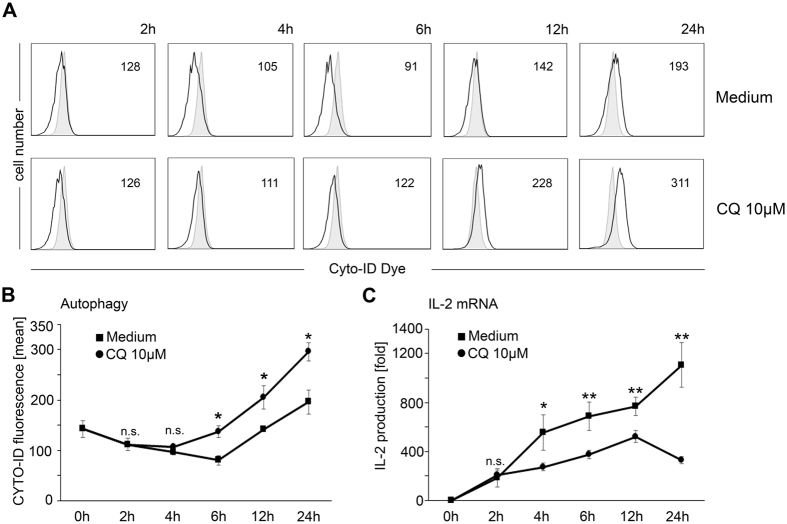
Time course analyses of autophagy and IL-2 production in CQ-exposed T-cells. (**A**) CD4^+^ T-cells were activated in the presence or absence (Medium) of 10 μM CQ. At the indicated time points, incorporation of a cationic amphiphilic tracer dye (Cyto-ID dye) was measured by FACS. Black line: stimulated cells, grey filled histograms: unstimulated cells. Numbers in the plot indicate mean fluorescence intensity. One representative experiment is shown. (**B**) Cumulative data (mean ± SD) from Cyto-ID FACS analyses from three independent experiments from cells activated in the absence (squares) or presence (circles) of 10 μM CQ. (**C**) IL-2 mRNA levels from CD4^+^ T-cells activated in the absence (squares) or presence (circles) of 10 μM CQ. Data indicate mean ± SD (n = 3) from fold-levels compared to unstimulated cells (paired samples t-test). n.s. not significant, *p < 0.05; **p < 0.01.

Having established that CQ directly inhibits parameters of T-cell activation, we aimed to identify underlying molecular modes of action. Accumulating evidence indicates that autophagy processes play important roles in regulating T-cell activation^13^,^14^. Given the well-described inhibition of autophagic flux by CQ, we thus assessed the influence of CQ on autophagy in T-cells^13^. For that purpose, CD4^+^ T-cells were stained with a fluorescent tracer, which selectively enriches in autophagic vesicles and thereby allows their sensitive and selective detection^22^. Notably, following anti-CD3/anti-CD28 activation, a slight down-regulation of the signal was observed, which might be indicative for an increased autophagic flux. Within the first four hours after T-cell activation, no significant effect of CQ was detectable on this process (Fig. 5A,B). Starting at six hours post activation, increased accumulation of the fluorescent tracer in CQ-exposed T-cells was observed in comparison to T-cells activated in plain medium (mean 137 ± 11 vs 80 ± 9; p = 0.027) indicating an increase in autophagic vesicles due to the inhibition of autophagosome turnover. This effect became more pronounced at later time points after T-cell activation (Fig. 5B). Concomitant time course analysis of IL-2 mRNA levels, as a read-out for early T-cell activation, revealed that the suppressive effect of CQ hereby preceded its impact on autophagy. A significant inhibition of IL-2 production by CQ was already detected four hours after T-cell activation (p = 0.026, Fig. 5C). Consequently, we hypothesized that, in addition to its autophagy inhibitory properties, CQ might directly inhibit intracellular signaling pathways that activate the IL-2 promoter.

### Effects of CQ on intracellular signaling

**Figure 6:**
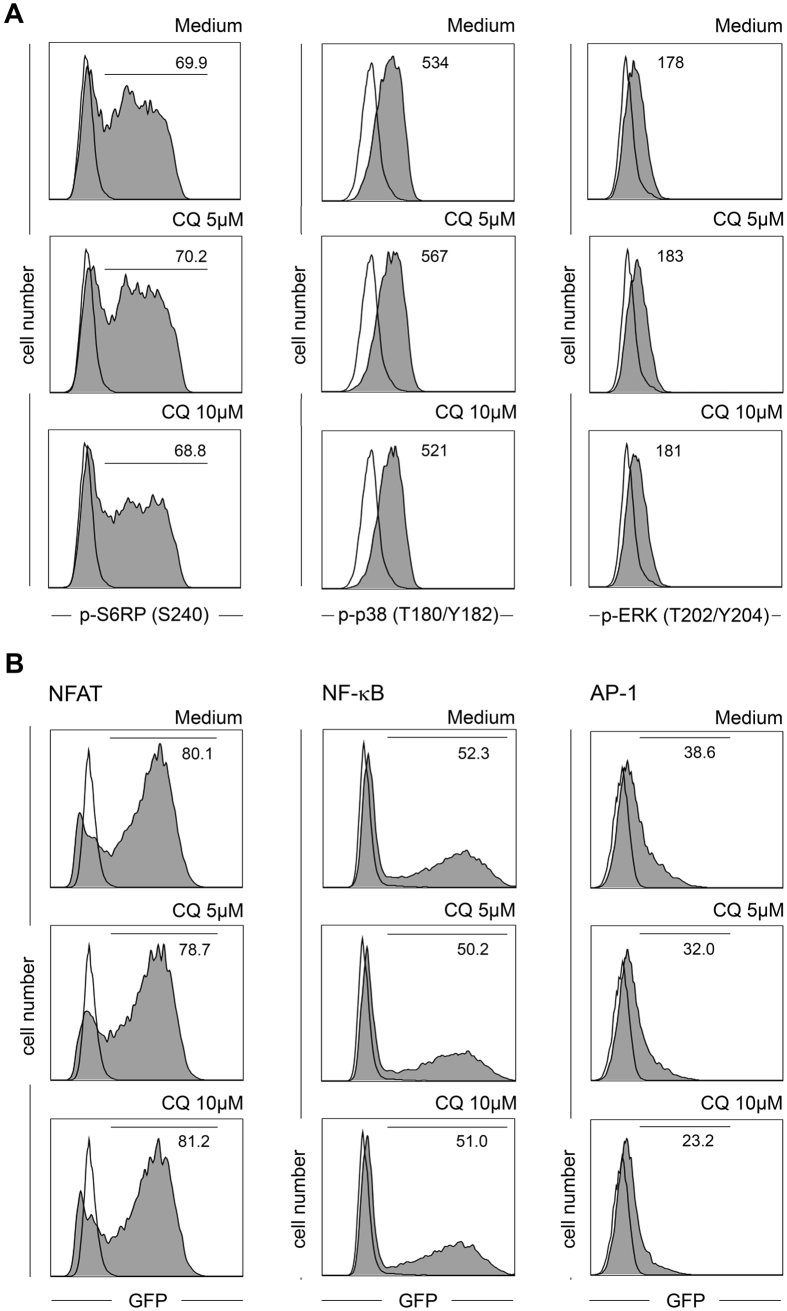
Intracellular signaling in CQ-exposed T-cells. (**A**) CD4^+^ T-cells were activated in the presence or absence (Medium) of the indicated concentrations of CQ. After 24 hours phosphorylation of S6RP, p38 and ERK was measured by FACS. Black line: unstimulated cells, grey histograms: stimulated cells. Numbers indicate percentage of positive cells (S6RP) or mean fluorescence intensity (p38 and ERK). One representative experiment (n = 6) is shown. (**B**) Jurkat T-cells expressing either an NFAT::GFP (left column), NF-κB::GFP (middle column) or AP-1::GFP (right column) reporter construct were activated with anti-CD3/CD80 expressing T-cell stimulator cells in the presence or absence (Medium) of CQ. After 24 hours GFP expression as readout for promoter activity was measured by FACS. Black line: unstimulated cells, grey histograms: stimulated cells. Numbers indicate percentage of positive cells. One representative experiment is shown (n = 6).

In a first step, we assessed major intracellular signaling nodes which are triggered following T-cell activation. Phosphorylation of the MAP-kinases ERK and p38 as well as the mTOR downstream target S6RP were not altered by CQ (p-value range: 0.344 − >0.999, Fig. 6A), indicating that TCR proximal signaling events and mTOR signaling are not influenced. Therefore, we analyzed the status of the three cardinal transcription factors NFAT, NF-κB and AP-1 using Jurkat reporter cells expressing either GFP reporter construct under control of the respective promoter elements^23^. At concentrations which were highly suppressive for the activation of primary T-cells (5 μM and 10 μM), CQ did not alter NFAT and NF-κB signaling in these Jurkat reporter cells (Fig. 6B). In contrast, down-regulation of the AP-1 reporter activity was consistently found following exposure to CQ. At 10 μM CQ, this inhibition amounted to 32.7% in comparison to cells activated in CQ-free medium (p < 0.001). Similar effects were also observed using the recently described triple parameter Jurkat reporter cell line^24^ (data not shown).

### Effects of CQ on AP-1 activation in primary CD4^+^ T-cells

**Figure 7:**
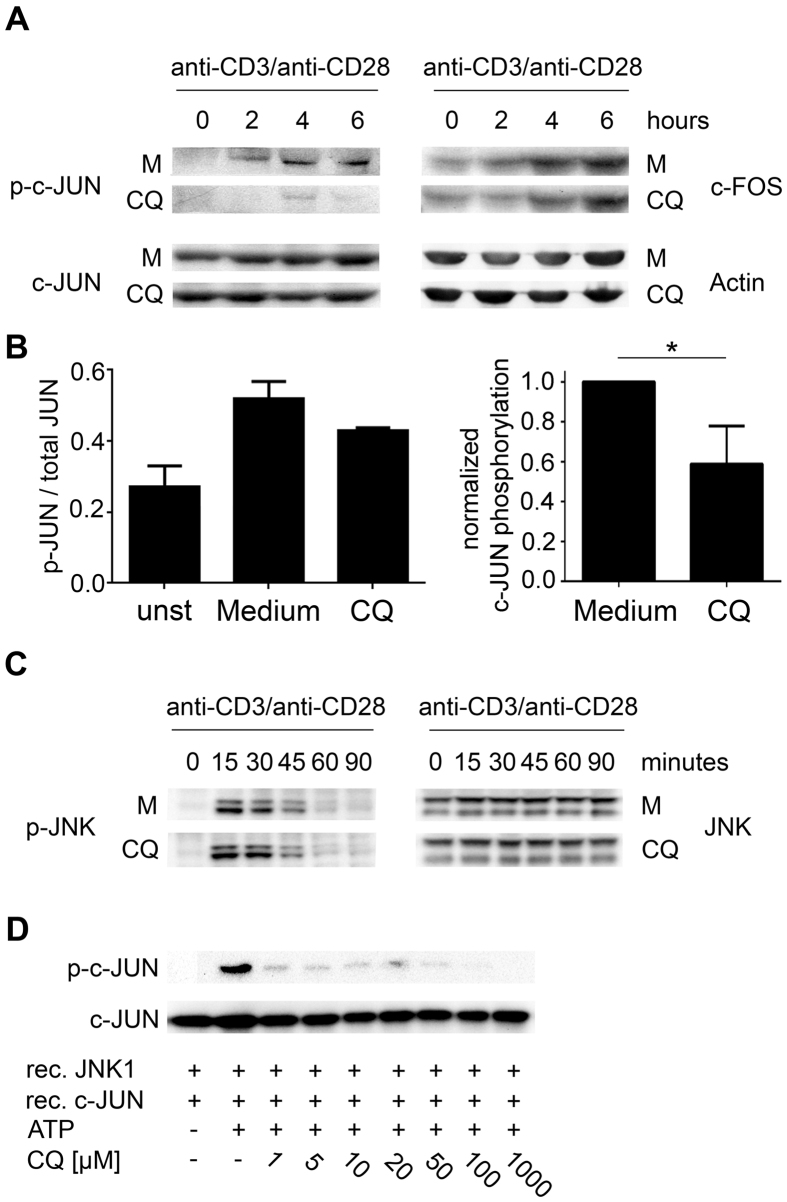
Effect of CQ on AP-1 activity in T-cells. (**A**) CD4^+^ T-cells were activated in the presence or absence (Medium; M) of 10 μM CQ for the indicated times. Phosphorylation of c-JUN (Ser73) as well as total levels of c-JUN and c-FOS were analyzed by immunoblotting. Expression of Actin served as loading control. One representative experiment (n = 3) is depicted. Different expositions from the same gel were cropped for presentation. (**B**) Analysis of c-JUN (Ser73) phosphorylation by ELISA. T-cells were activated in the presence or absence (Medium) of 10 μM CQ for 4 hours. For each condition, a ratio between the total protein signal and the phospho-protein signal (left panel) was calculated. Data show mean + SD from quintuplet measurements from one representative donor. Right panel, normalized induction of c-JUN phosphorylation after subtraction of values of unstimulated cells. Cumulative data of four donors. (**C**) CD4^+^ T-cells were activated in the presence or absence (Medium; M) of 10 μM CQ for the indicated time points. Phosphorylation of JNK (Thr183/Tyr185) as well as total levels of JNK were analyzed by immunoblotting, depicted plots were cropped from the same gel. One representative experiment (n = 3) is depicted. (**D**) *In vitro* phosphorylation of recombinant c-JUN by recombinant JNK1 in the presence or absence of CQ (1–1000 μM). Phosphorylated c-JUN (Ser73) and total c-JUN were detected by immunoblotting; depicted blots were cropped from the same gel. One representative experiment (n = 3) is shown. *p < 0.05.

Given the selective inhibition of AP-1 signaling by CQ in Jurkat T-cells, we further evaluated this effect in primary CD4^+^ T-cells. Main mechanisms of AP-1 activation in T-cells include the phosphorylation of c-JUN by JNK^25^ as well as the induction of c-FOS via the ERK-ELK-1 pathway^26^,^27^. Consequently, total levels of c-JUN and c-FOS and phosphorylation of c-JUN were assessed. A robust phosphorylation of c-JUN at Ser73 was observed 2 hours after T-cell activation in CQ-free medium and reached its peak after 4 to 6 hours. In contrast, no phosphorylation of c-JUN was detectable in CQ-exposed T-cells 2 hours after activation and only a slight signal could be detected at later times. In contrast to phospho-protein levels, no effect of CQ on total protein levels of c-JUN and c-FOS was observed (Fig. 7A and Supplementary Table I). To confirm these observations, we further performed a phospho-JUN specific ELISA. Phosphorylation of c-JUN was significantly decreased in CQ-exposed T-cell lysates compared to lysates of T-cells activated in CQ-free medium (normalized mean 0.59 ± 0.19, p = 0.009) (Fig. 7B). Since phosphorylation of c-JUN is mainly mediated by the kinase activity of JNK, we also measured the phosphorylation status of JNK by immunoblotting. However, JNK phosphorylation was not affected by CQ (Fig. 7C). Accordingly, we concluded that CQ might inhibit the catalytic function of JNK in signal transduction following activation. To confirm this hypothesis, we performed an *in vitro* kinase assay measuring phosphorylation of c-JUN by recombinant active JNK1. Addition of CQ strongly reduced the catalytic activity of JNK1 in a concentration range which is found in lymphocytes of patients under standard therapy^28^. JNK activity was completely abrogated at 1 mM CQ (activity < 1%; p < 0.001; Fig. 7D and Supplementary Figure III) and even at 1 μM CQ, a 87.1% reduction in c-Jun phosphorylation was observed, indicating that CQ acts as a highly potent inhibitor of JNK1 catalytic activity.

## Discussion

In this study we identified new immunomodulatory properties of CQ, a highly effective DMARD widely used in the therapy of SLE, RA and other connective tissue diseases^3^,^4^,^5^,^6^,^7^. Using concentrations mimicking intra-cellular drug levels of patients under CQ standard therapy^28^,^29^, exposure to CQ inhibited various activation parameters of T-cells, except for expression of the surface molecules CD25, CD69 and CD71. Regarding the pathophysiological role of Th subsets in various diseases^30^, knowledge about the sensitivity of Th subsets to immunosuppressive substances might be relevant for therapeutic purposes. In this respect, Th2 cytokine secretion was less sensitive to the inhibitory effects of CQ compared to cytokine secretion by Th1 and Th17 cells. In equimolar doses, HCQ had similar effects on T-cell activation (data not shown).

Immunomodulatory effects on activation induced T-cell functions, including cell proliferation, cytokine secretion but also upregulation of cell metabolism parameters, were found at concentrations between 0.6 and 10 μM CQ, while reduced cell viability was observed upon exposure to higher concentrations. Thus, suppressive effects were observed at lower concentrations than in previous mechanistic studies on T-cells and T-cell lines using 100 μM to 10 mM CQ^19^,^20^,^31^. However, the concentrations used by us perfectly mimic cellular concentrations reached during anti-rheumatic therapy^28^,^29^. Thus, our data should appropriately describe the modulatory capacity of CQ in patients.

Studies showing that activated T-cells accumulate autophagic vesicles and knockout of autophagy genes impairs T-cell effector functions have indicated a possible relationship between autophagy and T-cell functions^13^,^14^. Since CQ acts as inhibitor of autophagic flux, it is likely that these properties contribute to the immunosuppressive effect. Indeed, we found that CQ exposure led to an increased accumulation of autophagic vesicles in T-cells. Noteably, parallel time course analyses of autophagy and IL-2 mRNA expression as early activation read-out showed, that the inhibitory effect of CQ on IL-2 production preceded its effect on autophagy. Therefore, we hypothesized that the immunosuppressive function of CQ might not be solely restricted to the inhibition of autophagic flux and that CQ additionally affects signaling pathways which activate the IL-2 promoter.

In this respect, the best defined transcription factors are NFAT, NF-κB and AP-1, which also regulate other effector functions of activated T-cells^32^. The recent development of specific Jurkat reporter cell lines allows to screen the influence of manipulations on each of the above mentioned factors^23^,^24^. Using these tools, a selective inhibition of AP-1 signaling was observed, while NFAT and NF-κB signaling were unaffected. In T-cells, AP-1 activity is mainly regulated by the transcription of c-FOS and the phosphorylation status of c-JUN^25^,^26^,^27^ which is preferentially phosphorylated by JNK. Exposure of peripheral blood T-cells to CQ left induction of c-FOS protein and JNK phosphorylation fully intact, while phosphorylation of c-JUN was significantly decreased. In line with these results, *in vitro* analyses revealed that CQ directly inhibits enzymatic activity of recombinant JNK1. This effect was pronounced even at concentrations which are below intracellular levels reached under standard therapy. Given the chemical 4-amino-quinoline structure of CQ, our observations fit with the description of JNK inhibitors, which have a substituted quinoline structure^33^. The suppressive function of CQ on signaling pathways appears restricted to AP-1, since other important signaling nodes such as the MAP kinases ERK and p38 as well as the mTOR pathway were not altered.

The results of our study provide new insights into basic biological effects of JNK/AP-1 signaling in T-cell functionality. To our knowledge, this is the first report on primary human T-cells to indicate that up-regulation of cell surface activation markers might be differentially regulated from other T-cell activation parameters including proliferation, cytokine secretion and up-regulation of cell metabolism. Especially regulation of the latter in CD4^+^ T-cells is not fully understood to the day and several signaling pathways including mTOR and c-Myc are implied in these processes^34^,^35^. Our data strongly suggest that JNK/AP-1 signaling serves as additional module for the reprogramming of cell metabolism in activated T-cells. Moreover, these observations also concur with observations demonstrating that T-cell effector functions are regulated by distinct signaling modules in CD4^+^ T-cells^21^,^36^.

Different Th subsets might differentially utilize JNK/AP-1 signaling to produce specific effector cytokines. Especially secretion of IL-4 and IL-13 by Th2 cells showed less sensitivity to inhibition by CQ, suggesting a lower threshold of AP-1 activity for these processes. Notably, IL-5 production had the highest sensitivity to CQ exposure, indicating that IL-5 production in Th2-cells is differentially regulated in comparison to other Th2 cytokines. This hypothesis is supported by studies on murine Th2 cells that demonstrate that AP-1 is crucially involved in the regulation of IL-5 production^37^. Finally, anergic T-cells lack AP-1 signaling activity^38^. Since the effects of CQ were reversible in re-stimulation experiments, lack of JNK/AP-1 signaling in T-cells might be involved in the maintenance but not the induction of anergy. Thus, CQ acts differently to other exogenous^39^,^40^ and endogenous^21^ immunosuppressive substances, which induce states of hyporesponsiveness or anergy upon activation.

Our findings might also be relevant for novel clinical applications in T-cell-mediated diseases. CQ suppresses T-cell functions in a non-toxic and reversible manner and strongly affects cytokine secretion of Th1 and Th17 cells. Accordingly, in clinical conditions triggered by Th1 or Th17 cells, such as multiple sclerosis or inflammatory bowel diseases, CQ might have potential as a disease-modifying drug^41^,^42^. Moreover, CQ could also be applied in the therapy of malignant diseases with deregulated AP-1 pathway activity, as found in non-small cell lung cancers, colorectal or breast cancers^43^,^44^,^45^,^46^,^47^. Therefore, it remains to be investigated whether the AP-1 inhibitory potency of CQ contributes to the mode of action in cancer cells.

In this respect, it also needs to be determined whether potential direct effects of CQ on tumor cells are counteracted by the concomitant suppression of anti-tumor immunity. On the other hand, CQ might be beneficial tumorigenic processes, which are triggered by chronic inflammation. A plethora of clinical trials currently evaluating therapeutic effects of CQ in patients with atherosclerosis, autoimmune hepatitis, osteoarthritis, as well as colorectal cancer, ductal carcinoma, lung cancer, glioblastoma and multiple myeloma, could help shed light on the above-mentioned issues.

In conclusion, we provide a comprehensive overview of direct effects of CQ on T-cell activation. CQ inhibits activation-induced proliferation, metabolic activity and cytokine secretion in a reversible manner, whereas expression of activation markers is not affected. On the molecular level we defined inhibition of JNK/AP-1 signaling as a novel mode of action, which contributes to the immunosuppressive properties of CQ.

## Materials and Methods

### Ethical considerations, cell isolation and culture

The study was approved by the ethics committee of the Medical University of Vienna (EC number 1381/2014) and conducted according to the Declaration of Helsinki (1964, including current revisions) of the World Medical Association. Informed consent was obtained from all subjects. Peripheral blood mononuclear cells (PBMC) were isolated from healthy volunteers by Ficoll centrifugation (700 × g, 25 min, RT) and CD4^+^ T-cells (purity >95%) were isolated using the Magnisort CD4 Human T-cell Enrichment Kit (eBioscience, San Diego, CA). All functional assays were performed in IMDM + GlutaMax (GE Healthcare, Piscataway, NJ) supplemented with 10% fetal calf serum (GE Healthcare), 15 μg/mL gentamycin and 1 μg/mL Amphotericin B (both Sigma Aldrich, St. Louis, MO).

For all assays cells were pre-incubated with CQ (Sigma Aldrich) for 60 minutes and results were compared to cells without exposure to CQ. *In-vitro* levels were chosen to mimic intra-cellular drug concentrations found in patients under standard therapy with 400 mg CQ per day^28^,^29^. In all assays, cells were activated with anti-CD3/anti-CD28 coated microbeads (Invitrogen, Carlsbad, CA) at a cell : bead ratio of 2:1.

### Proliferation assays

1 × 10^5^ CD4^+^ T-cells were seeded in triplicates in 96-well flat bottom plates and activated after exposure to CQ. After 72 hours, cultures were pulsed with [methyl-^3^H] thymidine (1 μCi per well, Perkin Elmer, Boston, MA) for an additional 18 hours before being processed on a Packard scintillation counter (Packard, Meriden, CT)^21^,^23^. In some experiments Th1 cells (CD4^+^CD45RO^+^CXCR3^+^), Th2 cells (CD4^+^CD45RO^+^CXCR3^-^CCR6^-^CCR4^+^) and Th17 cells (CD4^+^CD45RO^+^CXCR3^-^CCR6^+^CCR4^+^)^48^ were isolated by flow cytometry and processed as above. The following antibodies were used for identification of these subsets: anti-human CD4 FITC (clone SK3), CD45RO eFluor450 (clone UCHL1), CXCR3 PE (clone CEW33D), CCR6 PerCP-Cy5.5 (clone R6H1) and CCR4 APC (clone D8SEE; all eBioscience). For re-stimulation experiments, 1 × 10^6^ CD4^+^ T-cells were activated in the presence or absence of CQ. After seven days, cells were harvested and washed extensively in CQ-free IMDM, and 1 × 10^5^ viable cells were re-seeded in triplicates. Cells were then activated for another 72 hours and proliferation was measured as described above.

As a second read-out for proliferation, CD4^+^ T-cells were labelled with 1.5 μM eFluor670 cell proliferation dye (eBioscience) prior to exposure to CQ. In these assays, proliferation was measured by determining the dilution of the CPD on a FACS Canto II flow cytometer (BD, Franklin Lakes, NJ). For comparison, a division index was calculated using the following formula^49^:


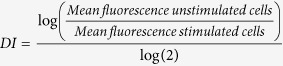


### Bioenergetic assays

Analyses of OCR (Oxygen Consumption Rate) and ECAR (Extracellular Acidification Rate) were performed using the XF24 Extracellular Flux Analyzer (Seahorse Bioscience, North Billerica, MA, USA)^21^. In brief, T-cells (4 × 10^6^) were activated in the presence or absence (medium) of CQ. After 48 hours cells were harvested, washed once in PBS and seeded in XF 24-well cell culture microplates (1 × 10^6^/well) pre-coated with Cell-Tak (Seahorse Bioscience) for 2 hours. Cells were maintained in a CO_2_-free incubator at 37 °C for one hour prior to the assay. At the indicated times OCR/ECAR levels were measured for three minutes. For the mitochondrial stress test, the ATP synthase inhibitor Oligomycin (1 μM) was added, followed by 3 μM FCCP to induce mitochondrial uncoupling. Non-mitochondrial respiration was measured after injection of Rotenone/antimycin A (1 μM each).

### Cytokine secretion assays

After 24 hours (IL-2) and 72 hours (IL-4, IL-13, IL-17, IFN-γ) of T-cell activation, supernatants were collected and cytokine levels were determined by Luminex multiplex analyses (Luminex Corporation, Austin, Texas). To determine effector cytokine secretion of Th subsets, supernatants of FACS-sorted Th subsets (see above) were collected 72 hours after activation. The secretion levels of IL-4, IL-5, IL-13, IL-17 and IFN-γ were measured using Luminex multiplex analyses.

### Cell viability assays

1 × 10^6^ CD4^+^ T-cells were activated in the presence of the indicated concentrations of CQ. After 72 hours, cells were harvested and washed once in Annexin staining buffer (10 mM HEPES, 140 mM NaCl, 2.5 mM CaCl2, pH 7.4, all Sigma Aldrich). Samples were incubated with 5 μL Annexin V-APC (eBioscience) for 10 minutes at room temperature, followed by washing in Annexin staining buffer and addition of propidium iodide (PI, Sigma Aldrich; final concentration of 100 ng/mL). Annexin V^−^/PI^−^ cells in cultures were identified as viable cells by flow cytometry.

### Flow cytometry

5 × 10^5^ CD4^+^ T-cells were activated in the presence or absence of CQ. Unstimulated cells and cells activated in drug-free medium (positive control) were included as controls. After 24 hours, cells were harvested, washed with PBS + 0.5% BSA + 0.05% NaN_3_ (all Sigma Aldrich) and incubated with anti-human CD25-PE-Cy7 (clone: BC96), CD69-APC (clone: FN50) and CD71-PE (clone: OKT9, eBioscience).

For measurement of autophagy-associated intracellular vacuoles, we used the Cyto-ID Autophagy Detection Kit (Enzo Life Science, Loerrach, Germany) according to the manufacturers’ instructions. 5 × 10^5^ CD4^+^ T-cells were activated in the presence or absence of CQ. Unstimulated cells and cells activated in drug-free medium (positive control) were included as controls. At the indicated time points, cells were harvested, washed once in staining buffer and were incubated with a cationic amphiphilic tracer dye for 20 minutes at room temperature. After washing incorporation of the dye into autophagic vacuoles was quantified by flow cytometry.

To determine phosphorylation of intracellular signaling proteins, cells were activated as above (10 μM CQ) and processed as described previously^21^,^23^. Cells were harvested and fixated in Fixation Buffer I (BD Phosflow, BD Biosciences) at 37 °C for 10 minutes. After washing in PBS + 0.5% BSA + 0.05% NaN3, cells were re-suspended in pre-chilled (−20 °C) Permeabilization Buffer III (BD Phosflow) and incubated on ice for 30 minutes. Afterwards, cells were washed twice in PBS + 0.5% BSA + 0.05% NaN_3_ and 20 μL anti-phospho-S6RP (S240; Alexa Fluor 647 conjugated; clone N4-41), anti-phospho-ERK (T202/Y204; Pacific Blue conjugated; clone 20 A) and anti-phospho-p38 (T180/Y182; PE conjugated; clone 36/p38; BD Phosflow) or isotype-matched control antibodies were added for 60 minutes. Subsequently, cells were washed and analyzed on a FACS Canto II flow cytometer.

### Quantitative PCR

For relative quantification of RNA production, T cells were activated in the presence or absence of 10 μM CQ. At the indicated time points, RNA was isolated using a QIAcube RNA isolation station (Qiagen, Hilden, Germany), and cDNA was generated by random hexamer-primed reverse transcription. Relative transcriptional levels of IL-2 were quantified using the iTaq SYBR Green Supermix (Bio-Rad, Hercules, CA, USA) on a 7900HT Fast Real-Time PCR system (Applied Biosystems, Foster City, CA, USA). Transcriptional levels of beta-2-microglobulin were used as reference. The following primers were used: IL-2 for: 5′-ATGAGACAGCAACCATTGTAGAATTT-3′, IL-2 rev: 5′-CACTTAATTATCAAGTCAGTGTTGAGATGA-3′, b2m for: 5′-GGAATTGATTTGGGAGAGCATC-3′, b2m rev: 5′-CAGGTCCTGGCTCTACAATTTACTAA-3′. For quantification ΔCT values from the respective samples were calculated (ΔCT = CT_IL-2_ − CT_b2m_) and fold-expression of IL-2 was calculated according to the formula


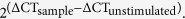


### NFAT, NF-κB and AP-1 reporter assays

1 × 10^6^ Jurkat T-cells expressing an NFAT::GFP, NF-κB::GFP or AP-1::GFP promoter element were stimulated with 2 × 10^5^ BW 5147 T-cell stimulator cells transduced with a membrane-bound OKT3 single-chain fragment variable and human CD80 (TCS CD80)^50^ in the presence or absence of 5 μM or 10 μM. Unstimulated reporter cells served as a reference. After 24 hours, cells were harvested and the percentage of GFP-positive cells was established by flow cytometry. Promoter activity was calculated relative to the percentage of positive cells from samples activated in substance-free medium^21^.

### Immunoblotting

4 × 10^6^ CD4^+^ T-cells were activated in the presence or absence of 10 μM CQ for the indicated times. Cells were lysed in RIPA buffer with protease and phosphatase inhibitors (Sigma Aldrich). Cellular debris was removed (25,000 × g, 4 °C, 15 minutes). Samples were resolved by SDS-PAGE on 4–12% gradient gels under reducing conditions (Life Technologies, Paisley, UK), followed by transfer onto PVDF membranes (GE Healthcare). For immunoblotting the following antibodies were used: rabbit anti-p-c-JUN (Ser73; clone D47G9), mouse anti-c-JUN (clone L70B11), rabbit anti-c-FOS (clone 9F6), rabbit anti-Actin (clone D18C11), rabbit anti-phospho-SAPK/JNK (Thr183/Tyr185; clone 81E11), rabbit anti-SAPK/JNK (clone 56G8; all New England Biolabs, Ipswich, MA). After incubation with secondary horseradish peroxidase-conjugated antibodies, binding was visualized using the SuperSignal West Pico Chemiluminescent Substrate (Thermo Scientific, Rockford, IL). To allow statistic evaluation of the immunoblots, band intensity was quantified using the ImageJ software (National Institute of Health, Bethesda, MD; Version 1.50e).

### Phospho-c-JUN ELISA

The (total/phospho) InstantOne ELISA (eBioscience) was used according to the manufacturer’s instructions. In brief, 4 × 10^6^ CD4^+^ T-cells were activated in the presence or absence of 10 μM CQ. After 4 hours, cells were harvested, lysed and protein concentrations in the lysates were adjusted to 0.5 μg/μL. Lysates were incubated for one hour with either a total c-JUN capture/detection antibody mix or a phospho c-JUN capture/detection antibody mix in quadruplets. Afterwards samples were washed and incubated in substrate solution and absorbance was measured at 450 nm. A phospho : total absorbance ratio was calculated from each sample.

### *In vitro* JNK activity assay

The SAPK/JNK activity assay kit (New England Biolabs) was used according to the manufacturers’ instructions. Recombinant human JNK1 (5 ng) was incubated with CQ (1 μM-1 mM) or plain reaction buffer at room temperature for 15 min. The kinase reaction was initiated by the addition of recombinant human c-JUN (1 μg) and ATP (200 μM). Phosphorylation of the c-JUN target peptide was conducted by incubation of the reaction mix at 30 °C for 30 min. The negative control reaction was performed in the absence of ATP. The reaction was stopped by addition of SDS sample buffer and incubation at 98 °C for 15 minutes. Afterwards, samples were resolved on by SDS-PAGE, transferred to a PVDF membrane and detection of phosphorylated and total c-JUN was performed as described above.

### Statistical analysis

Statistical analyses were performed using GraphPad Prism (GraphPad Software Inc., La Jolla, CA, Version 6.07). Proliferation rates, cell viability and cytokine levels were normalized to corresponding controls using fixed point normalization as evaluated by *Degasperi et al*.^51^ and are given in mean percentages ± standard deviation.

For group comparison, an analysis of variance (ANOVA) with repeated measures was performed. For post-hoc testing, Dunett’s test (comparison to medium) or the Holm-Šídák test (multiple comparisons between subgroups) was performed using adjusted p-values. Statistical significance was defined as a p-value < 0.05 (two-sided). In figures, statistically significant values are depicted as follows: *p < 0.05; **p < 0.01; ***p < 0.001.

## Additional Information

**How to cite this article**: Schmidt, R. L. J. *et al*. Chloroquine inhibits human CD4^+^ T-cell activation by AP-1 signaling modulation. *Sci. Rep.* **7**, 42191; doi: 10.1038/srep42191 (2017).

**Publisher's note:** Springer Nature remains neutral with regard to jurisdictional claims in published maps and institutional affiliations.

## Supplementary Material

Supplementary Information

## Figures and Tables

**Table 1:** Cytokine levels measured in CD4^+^ T-cells.

Cytokine	ANOVA^1^	Medium	CQ 5 μM^2^	CQ 10 μM^2^
IL-2	n = 6	1.00 ± 0.00	0.63 ± 0.06	0.55 ± 0.10
	p < 0.001	—	p < 0.001	p < 0.001
IL-10	n = 8	1.00 ± 0.00	0.63 ± 0.22	0.30 ± 0.15
	p < 0.001	—	p < 0.001	p < 0.001
TNF-α	n = 5	1.00 ± 0.00	0.75 ± 0.16	0.62 ± 0.18
	p = 0.008	—	p = 0.042	p = 0.015
IFN-γ	n = 12	1.00 ± 0.00	0.52 ± 0.17	0.38 ± 0.15
	p < 0.001	—	p < 0.001	p < 0.001
IL-4	n = 6	1.00 ± 0.00	0.95 ± 0.19	0.59 ± 0.20
	p = 0.001	—	p = 0.777	p = 0.007
IL-13	n = 7	1.00 ± 0.00	0.89 ± 0.19	0.63 ± 0.13
	p < 0.001	—	p = 0.379	p = 0.001
IL-17	n = 11	1.00 ± 0.00	0.67 ± 0.07	0.32 ± 0.06
	p < 0.001	—	p < 0.001	p < 0.001

Data is given as mean ± SD.

^1^ANOVA: analysis of variance with repeated measures for global testing.

^2^post-hoc testing: Dunett’s test.
