# Supplementary material for: Chloroquine inhibits human CD4+ T-cell activation by AP-1 signaling modulation
Source: Sci Rep. 2017 Feb 7;7:42191. doi: 10.1038/srep42191 (PMC5294581; doi:10.1038/srep42191)
Supplement: Supplementary Information [file srep42191-s1.pdf]

Supplementary Information

Chloroquine inhibits human CD4+ T-cell activation by AP-1 signaling modulation

Ralf L.J. Schmidt, Sabrina Jutz, Katrin Goldhahn, Nadine Witzeneder, Marlene C. Gerner, Doris Trapin, Georg Greiner, Gregor Hoermann, Guenter Steiner, Winfried F. Pickl, Heinz Burgmann, Peter Steinberger, Franz Ratzinger and Klaus G. Schmetterer

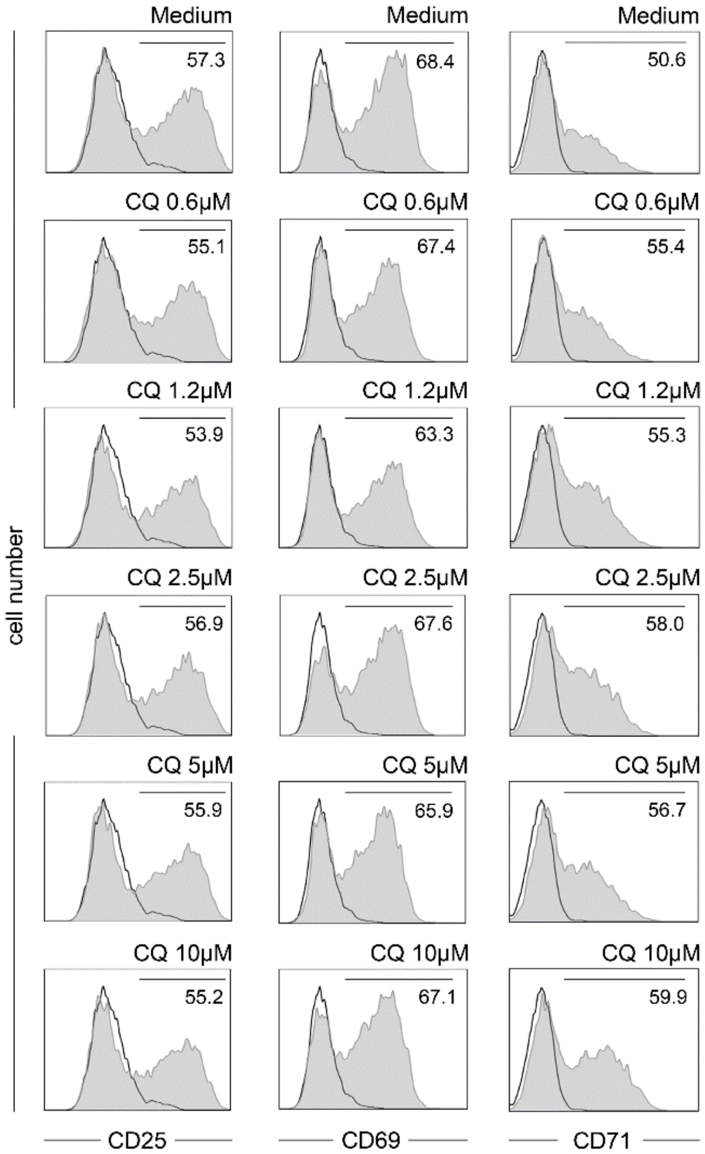

Supplementary Figure I. Effect of CQ on activation-marker expression on $CD4^{+}$ T-cells; representative histograms of T-cells activated in the presence or absence (Medium) of CQ. Black line: unstimulated cells; grey filled histograms: cells activated for 24 hours under the indicated condition. Numbers indicate percentage of positive cells.

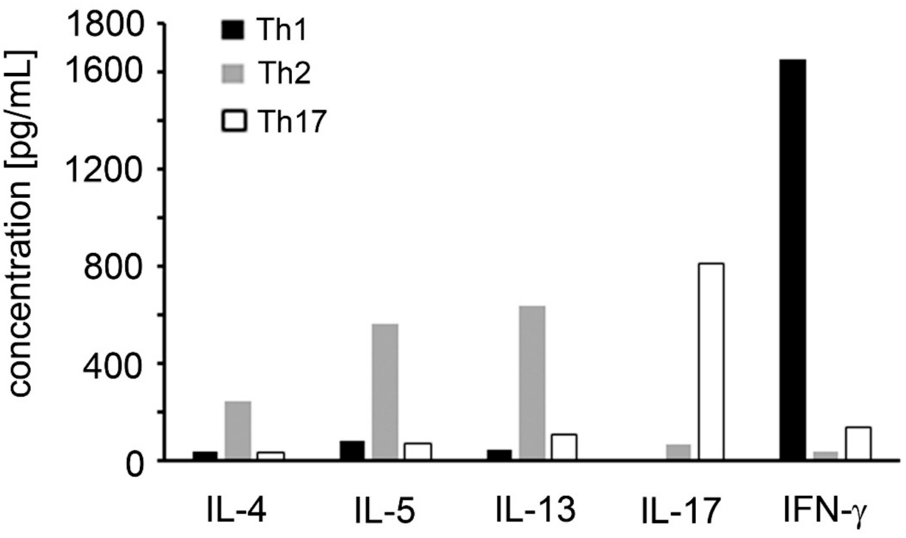

Supplementary Figure II. Cytokine secretion by FACS-sorted Th cells; secretion of IL-4, IL-5, IL-13, IL-17 and IFN-γ by Th1 cells (black bars), Th2 cells (grey bars) and Th17 cells (white bars) sorted according to the chemokine receptor profile as outlined in the Materials and Methods section. Data show mean values from duplicate measurements from one representative donor (n=4).

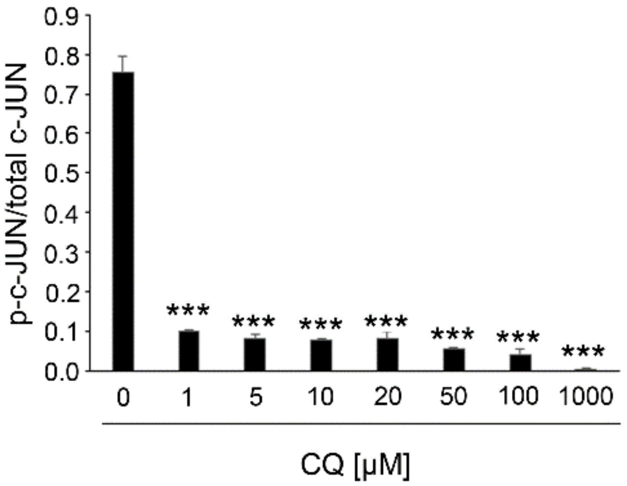

Supplementary Figure III. Cumulative data from in vitro JNK activity assay; for each sample band intensity was determined by densitometry and the ratio between phosphorylated c-JUN and total c-JUN was calculated. Data depict mean + SD from three independent experiments. *** $p<0.001$

**Immunoblot**	Medium	CQ 10µM	p-value
**p-c-JUN/total c-JUN**			0.006[a]
0h	0.06 ± 0.03	0.05 ± 0.01	0.633[b]
2h	0.44 ± 0.05	0.08 ± 0.04	0.001[b]
4h	0.68 ± 0.06	0.16 ± 0.02	<0.001[b]
6h	0.77 ± 0.03	0.24 ± 0.03	<0.001[b]
**total c-JUN/Actin**			0.436[a]
0h	1.03 ± 0.03	1.04 ± 0.04	0.721[b]
2h	1.00 ± 0.01	1.01 ± 0.02	0.413[b]
4h	0.99 ± 0.01	1.04 ± 0.05	0.223[b]
6h	1.01 ± 0.02	1.02 ± 0.01	0.429[b]
**c-FOS/Actin**			0.540[a]
0h	0.33 ± 0.04	0.36 ± 0.07	0.712[b]
2h	0.63 ± 0.01	0.61 ± 0.04	0.429[b]
4h	0.85 ± 0.01	0.84 ± 0.05	0.740[b]
6h	1.00 ± 0.02	1.01 ± 0.01	0.825[b]

Supplementary Table I. Statistical analysis from AP-1 immunoblotting; Signal intensities at the indicated time points after T-cell activation were determined by densitometry, corrected for background intensity and the indicated ratios were calculated. Mean ± standard deviation from three independent experiments is shown.

[a]one-way ANOVA with repeated measures, [b]Dunnett's multiple comparisons test
